# Ruxolitinib discontinuation syndrome: incidence, risk factors, and management in 251 patients with myelofibrosis

**DOI:** 10.1038/s41408-020-00392-1

**Published:** 2021-01-07

**Authors:** Francesca Palandri, Giuseppe Alberto Palumbo, Elena Maria Elli, Nicola Polverelli, Giulia Benevolo, Bruno Martino, Elisabetta Abruzzese, Mario Tiribelli, Alessia Tieghi, Roberto Latagliata, Francesco Cavazzini, Micaela Bergamaschi, Gianni Binotto, Monica Crugnola, Alessandro Isidori, Giovanni Caocci, Florian Heidel, Novella Pugliese, Costanza Bosi, Daniela Bartoletti, Giuseppe Auteri, Daniele Cattaneo, Luigi Scaffidi, Malgorzata Monica Trawinska, Rossella Stella, Fiorella Ciantia, Fabrizio Pane, Antonio Cuneo, Mauro Krampera, Gianpietro Semenzato, Roberto Massimo Lemoli, Alessandra Iurlo, Nicola Vianelli, Michele Cavo, Massimo Breccia, Massimiliano Bonifacio

**Affiliations:** 1 Azienda Ospedaliero-Universitaria di Bologna, IRCCS Istituto di Ricovero e Cura a Carattere Scientifico, Policlinico S.Orsola-Malpighi, Dipartimento di Oncologia e di Ematologia, Bologna, Italia; 2 Department of Scienze Mediche, Chirurgiche e Tecnologie Avanzate “G.F. Ingrassia”, University of Catania, Catania, Italy; 3 Hematology Division, San Gerardo Hospital, ASST Monza, Monza, Italy; 4 Unit of Blood Diseases and Stem Cells Transplantation, Department of Clinical and Experimental Sciences, University of Brescia, ASST Spedali Civili of Brescia, Brescia, Italy; 5 Division of Hematology, Città della Salute e della Scienza Hospital, Torino, Italy; 6 Division of Hematology, Azienda Ospedaliera ‘Bianchi Melacrino Morelli’, Reggio Calabria, Italy; 7 Division of Hematology, Ospedale S. Eugenio, Roma, Italy; 8 Division of Hematology and BMT, Department of Medical Area, University of Udine, Udine, Italy; 9 Department of Hematology, Azienda USL - IRCCS di Reggio Emilia, Reggio Emilia, Italy; 10 Division of Cellular Biotechnologies and Hematology, University Sapienza, Roma, Italy; 11 Division of Hematology, University of Ferrara, Ferrara, Italy; 12 Clinic of Hematology, Department of Internal Medicine (DiMI), IRCCS AOU San Martino-IST, Genova, Italy; 13 Unit of Hematology and Clinical Immunology, University of Padova, Padova, Italy; 14 Haematology and BMT Centre, Azienda Ospedaliero-Universitaria di Parma, Parma, Italy; 15 Hematology and Stem Cell Transplant Center, AORMN Hospital, Pesaro, Italy; 16 Hematology Unit, Department of Medical Sciences and Public Health, University of Cagliari, Cagliari, Italy; 17 Internal Medicine II, Hematology and Oncology, Friedrich-Schiller-University Medical Center, Jena, Germany; 18 Department of Medicine and Surgery, Hematology and Hematopoietic Stem Cell Transplant Center, University of Naples Federico II, Napoli, Italy; 19 Division of Hematology, AUSL di Piacenza, Piacenza, Italy; 20 Hematology Division, Foundation IRCCS Ca’ Granda Ospedale Maggiore Policlinico, Milano, Italy; 21 Department of Medicine, Section of Hematology, University of Verona, Verona, Italy; 22 Division of Hematology, AOU Policlinico V. Emanuele, University of Catania, Catania, Italy

**Keywords:** Myeloproliferative disease, Haematological diseases

**Dear Editor**,

RRuxolitinib (RUX) is the first *JAK1/JAK2* inhibitor (JAKi) approved for the treatment of splenomegaly and symptoms related to myelofibrosis (MF)^1,2^. By *JAK1* inhibition, RUX reduces the production of several inflammatory cytokine (IL-6, IL-1rα, MIP-1β, TNF-β, and CRP), whereas myelosuppression is mainly exerted through *JAK2* inhibition. Despite considerable clinical efficacy, some patients fail to obtain and/or maintain a stable response or are intolerant to RUX^3,4^. Thus, ~40% of patients discontinue RUX within 3 years of therapy^5^.

In the early phase I/II study of RUX in MF, most patients experienced relapse of their symptoms and worsening splenomegaly after RUX discontinuation^1^, and life-threatening adverse events (AEs) occurred in 5 out of 47 patients, including respiratory distress, septic-like shock and disseminated intravascular coagulation-like syndrome. These events, attributed to an acute rebound of cytokine storm, were defined as RUX discontinuation syndrome (RDS), and careful tapering under close physician supervision was suggested as a preventive strategy^6^. Further cases of severe AEs attributed to RUX discontinuation have been subsequently described, despite a careful stepwise reduction of RUX^7–9^, also in the setting of patients that received RUX as a bridge to transplantation^10,11^ (Supplemental Table 1). RDS typically presents within 3 weeks from RUX discontinuation, apparently without relation with RUX dose, and seems to improve after RUX reintroduction. However, these findings were based on case reports or limited series of patients, and available data are insufficient to estimate the impact of RDS in routine clinical practice.

The current study aims to investigate in a real-world context: (1) modalities of RUX discontinuation; (2) incidence, timing, and severity of RDS; (3) outcome and risk factors associated with RDS.

In 2016, a clinical network was established to collect information about RUX therapy in MF^12^. This network now includes 22 academic hematology centers where MF patients are followed by hematologists with specific MPN-driven practice.

A specific survey was conducted in all participating Centers with the scope to obtain comprehensive information regarding timing/modalities of RUX discontinuation, and subsequent outcome. RDS included all new symptoms that occurred within 21 days from RUX discontinuation and were interpreted by the treating Hematologist as caused by RUX discontinuation. Based on the previous definition, RDS was graded as mild if no intervention was required, moderate if symptoms required medical interventions including RUX restarting, steroids, oral analgesics, and severe if intravenous medications, hospital admissions, splenectomy, or delaying of hematopoietic allogeneic transplantation (HCT) were needed^11^.

At data cutoff date (1 May 2020), 700 RUX-treated MF patients were included in the database. After a median follow-up from RUX start of 36.1 months, 251 (35.9%) patients discontinued RUX and were evaluable for RDS.

At the time of decision to stop RUX, 53% of these 251 patients were older than 70, 68.5% presented anemia and 45.8% had large splenomegaly (>10 cm below costal margin, BCM); 27.5% of patients had a MPN-10 Total Symptom Score > 20. Failure (lack/loss of response, or leukemic transformation) was the main cause of RUX stop (60.6%), whereas AEs (mainly hematological) and other reasons caused RUX discontinuation in 28.6% and 10.8% of patients, respectively. In most cases, AEs were concomitant to failure/suboptimal responses. Indeed, at the time of RUX stop, 36.7% of patients presented a platelet count below 100 × 10^9^/l and 31.5% had transfusion-dependent anemia. Also, in 7.6% of the patients, a grade 2–3 infectious event was recorded before RUX discontinuation.

RUX daily dose (mg BID) was: 5, 10, 15, or 20 in 46.2, 23.1, 15.6, and 15.1% of patients. Notably, at the time of RUX start, the dose of RUX was 5, 10, 15, or 20 mg BID in 27.9, 17.2, 18.4, and 36.5% of patients. The concomitance of AEs with a poor response to RUX most likely led to a progressive RUX dose reduction in many patients; hence, at the time of the final decision to discontinue the drug, many patients were already taking low doses.

In 162 patients, RUX was abruptly discontinued. In the remaining 89 patients (35.5%), RUX dose was gradually decreased before discontinuation. Tapering was associated with hydroxyurea and/or corticosteroids in 34 (38.2%) patients. RUX tapering pattern was very variable among Centers and consisted of dose reductions of 5 or 10 mg per day at variable intervals, ranging from a dose reduction every 30 days to one every 3 days. The median duration of tapering was 14 days (range 3–60). No association was found between tapering use and clinical/laboratory parameters or RUX dose at the time of RUX discontinuation. However, tapering use was consistent within the single Centers, and was regularly performed in only 4 Hematology Centers (Supplemental Fig. 1).

RDS occurred in 34 (13.5%) patients after a median time of 7 days (range, 2–21) from RUX stop. The incidence rate of RDS was 0.7 per 100 patient-days.

RDS was mild in 21 (61.8%) patients. Mild RDS consisted in symptomatic spleen increase in 62% of the cases, whereas 9.4% of patients experienced a flare in constitutional symptoms (fever, weight loss, night sweats); in six patients (28.6%), other MF-related symptoms (fatigue, itching, bone pain, abdominal discomfort) occurred. The median time from RUX stop to mild RDS was 10 days (3–21). After RUX discontinuation, ten patients (47.7%) did not receive further therapy because of progression to blast phase and/or unfitness; eight patients (38%) were treated with different therapies (including demethylating agents, splenectomy, and HCT), whereas three patients (14.3%) received a JAKi, after a median time of 9 months.

A moderate RDS occurred in 10 out of 34 (29.4%) patients, after a median time from RUX stop of 8.5 days (range, 3–20) and comparable to mild RDS. Moderate RDS was represented by symptomatic spleen enlargement (seven patients), or constitutional symptoms appearance/increase (three patients). RDS therapy consisted in corticosteroids (eight patients) or enrollment in a clinical trial with a non-JAKi. Three patients received RUX rechallenge after an average time of 2.6 years from first discontinuation.

A total of three cases of severe RDS were observed and consisted of: spleen rupture causing splenectomy (case 1); fever, dyspnea, confusion, and dizziness requiring hospitalization (case 2); severe ARDS treated in intensive care unit (case 3) (Supplemental Figure 2). Severe RDS occurred within 48 h after RUX discontinuation and the patient’s condition rapidly improved after RUX rechallenge.

No fatal cases of RDS were observed.

In multivariable Cox regression analysis, only platelet count <100 × 10^9^/l (HR 2.98, 95%CI 1.29–6.90) and spleen ≥10 cm BCM (HR 2.03, 95%CI 1.01–4.17) at RUX stop were significantly associated with higher probability of RDS (Fig. 1a). Overall, 19 out of 251 patients (7.6%) re-started RUX after drug discontinuation. RDS was significantly associated with the need of RUX rechallenge, with 8/34 (23.5%) RDS patients eventually resuming RUX (*p* < 0.001) (Fig. 1b). Notably, the occurrence of RDS did not significantly influence overall survival.

**Fig. 1:**
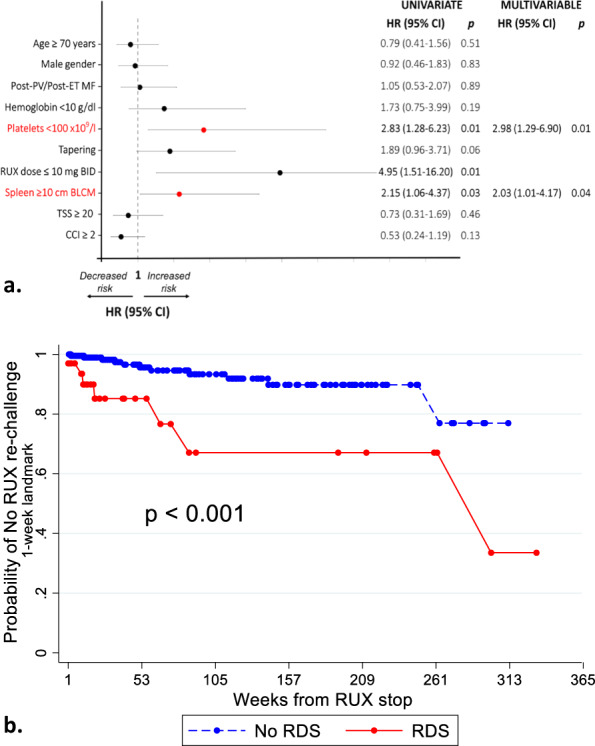
Risk factors at ruxolitinib discontinuation associated with subsequent discontinuation syndrome (a) and probability of RUX rechallenge according to RDS (b). **a** Risk factors were identified with a Cox regression model. In order to use a parsimonious model owing to the small number of events, only variables with *p* value < 0.05 in univariate analysis were considered for multivariable analysis. Moreover, collinearity amongst variables was detected by means of Pearson correlation test. Ultimately only platelet count and spleen size were considered in multivariable analysis and both remained statistically significant. **b** Given that RDS is a time-dependent covariate, the curves were obtained with the Simon-Makuch technique to take the change in an individual’s covariate status over time into account. One week from ruxolitinib stop, which is the median time from RUX stop to RDS occurrence, was chosen as the landmark time point.

Overall, this study shows that symptoms and/or splenomegaly significantly increase in ~15% of patients soon after RUX stop, with sometimes considerable clinical deterioration in already frail patients. This frequency is consistent with the report by Shanavas et al.^11^, who found that 10 out of 66 (15%) patients treated with RUX before HCT developed RDS. RDS was mild to moderate in eight cases, and severe in two cases, eventually leading to HCT delay. In our cohort, the incidence of severe RDS appeared to be lower (1%) than initially reported (11%)^6^. Whether this difference is related to the smaller number or to a more advanced disease of patients enrolled in phase I–II study, compared with those treated with RUX in subsequent years, remains to be defined. Indeed, compared with patients included in the phase I/II trial, that were all at intermediate-2/high risk and mostly (92%) affected by severe splenomegaly, this study included many patients that started RUX while at intermediate-1 risk (47.2%), with a lower incidence of baseline large splenomegaly (63.3%) or thrombocytopenia (15.4%). Also, owing to its retrospective nature, an underestimation of RDS cannot be fully ruled out in the present study. However, both experiences highlight that re-expansion of MF after RUX can be sudden and potentially life-threatening, and that a rapid diagnosis of RDS is critical, as the reintroduction of RUX can quickly improve clinical status in most cases.

Since RDS is an early event, any other therapy should be started as close as possible to RUX discontinuation, as already observed and indicated in the context of RUX as bridge to HCT^13^. Importantly, the possible occurrence of RDS may be anticipated in a substantial fraction of patients that discontinue RUX during the screening phase of clinical trials enrolling patients with failure or suboptimal response to RUX. In these cases, careful monitoring, and disclosure of potential risk of RDS to the patients are recommended.

This real-world experience also highlights that, despite specific indications^14^, prevention strategies of RDS were infrequent and inconsistent across different Centers, with only a minority of patients gradually reducing the dose or introducing prophylactic corticosteroids. This has probably prevented the detection of a correlation between tapering and RDS reduction. Despite these limitations, implementation and standardization of discontinuation strategies should be pursued for a better RDS prevention in the future.

Finally, we observed that the risk of RDS was significantly higher in patients with a greater burden of the disease at the time of discontinuation. Particularly, the increased incidence of RDS in patients with large splenomegaly may indicate that unexpectedly, in at least some patients deemed refractory to RUX, the maintenance of JAK2 inhibition has a non-negligible activity. In this context, the re-use of a JAKi may be particularly reasonable. The reintroduction of RUX has already shown to achieve some clinical responses^15^. Possibly, second-generation JAKi may have an even greater clinical relevance in this setting.

In conclusion, these results confirm the need for a careful surveillance of MF patients at the time of RUX discontinuation. A quick switch to alternative treatments, if clinically indicated, should be planned particularly for patients who stop RUX with large splenomegaly. In the absence of available alternatives, the occurrence of RDS may indicate a residual disease control activity and identify a population that can still benefit from JAK2i.

## Supplementary information

Supplemental Table 1

Supplemental Figure 1

Supplemental Figure 2
