# Supplementary material for: Ruxolitinib discontinuation syndrome: incidence, risk factors, and management in 251 patients with myelofibrosis
Source: Blood Cancer J. 2021 Jan 7;11(1):4. doi: 10.1038/s41408-020-00392-1 (PMC7791065; doi:10.1038/s41408-020-00392-1)
Supplement: Supplementary file 1 — Supplemental Table 1 [file 41408_2020_392_MOESM1_ESM.docx]

**Supplemental Table 1. Characteristics of published cases of severe Ruxolitinib Discontinuation Syndrome (RDS).** ARDS: acute respiratory distress syndrome. DIC: disseminated intravascular coagulation. HCT: allogeneic stem cell transplantation. AML: acute myeloid leukemia. AE: adverse events. PPV-MF: post polycythemia vera myelofibrosis. PMF: primary myelofibrosis. ET: essential thrombocythemia. n.r.: not reported. Notably, 3 cases of Tumor Lysis Syndrome (TLS) and 3 cases of cardiogenic shocks were disclosed with no further details within the JAK-ALLO clinical trial (not shown)^10^

| **Patient no.**  **[ref]** | **Sex,  age** | **Disease** | **time  to RDS** | **Spleen  at stop** | **Symptoms at stop** | **Toxicity at stop** | **reason  for stop** | **RUX dose  at stop** | **Taper** | **steroids** | **RDS type** | **RUX  re-start** | **outcome** |
| --- | --- | --- | --- | --- | --- | --- | --- | --- | --- | --- | --- | --- | --- |
| 1 [6] | F, 59 | PPV-MF | 14 days | progressive | progressive | severe anemia | response loss | 10 mg BID | yes | yes | respiratory distress,  splenomegaly | no | investigational  JAK2-i |
| 2 [6] | F, 69 | PPV-MF | 1 day | improved | improved | mild anemia | unrelated AEs | 15 mg BID | no | no | shock | yes | aggressive Lymphoma |
| 3 [6] | M, 44 | PPV-MF | 1 day | progressive | progressive | severe anemia | response loss | 20 mg BID | yes | no | ARDS, pleural effusions | yes | investigational JAK2-i after successful RUX stop |
| 4 [6] | M, 64 | PMF | 3 days | improved | improved | thrombocytopenia | thrombocytopenia | 25 mg BID | no | no | fever, splenomegaly, symptoms | yes | recurrence of RDS 2 yrs later investigational JAK2-i |
| 5 [6] | F, 56 | PPV-MF | 14 days | progressive | progressive | thrombocytopenia | response loss  & thrombocytopenia | 10 mg BID | yes | no | DIC, severe arthritis | no | n.r. |
| 6 [7] | F, 70 | PPV-MF | 21 days | improved | improved | thrombocytopenia | thrombocytopenia | 5 mg BID | no | no | fever, splenomegaly, TLS | no | n.r. |
| 7 [8] | M, 76 | PMF | 1 day | improved | improved | thrombocytopenia | thrombocytopenia | 15 mg BID | no | yes | ARDS, fever | no | n.r. |
| 8 [9] | F, 56 | ET | 2 days | n.r. | n.r. | none | AML | 10 mg BID | yes | no | ARDS, fever | yes | HCT for AML |
| 9 [11] | F, 64 | PPV-MF | <10 days | n.r. | n.r. | n.r. | planned HCT | n.r. | no | no | pulmonary infiltrates, rebound splenomegaly | yes | splenectomy & delay HCT |
| 10 [11] | F, 52 | PPV-MF | <6 days | n.r. | n.r. | n.r. | planned HCT | n.r. | no | no | ARDS, fever | no | delay HCT, dead after HCT |
