# Supplementary material for: Ruxolitinib discontinuation syndrome: incidence, risk factors, and management in 251 patients with myelofibrosis
Source: Blood Cancer J. 2021 Jan 7;11(1):4. doi: 10.1038/s41408-020-00392-1 (PMC7791065; doi:10.1038/s41408-020-00392-1)

Supplemental Figure 1. Frequency of ruxolitinib (RUX) tapering at discontinuation in each of the 21 Hematology Centers

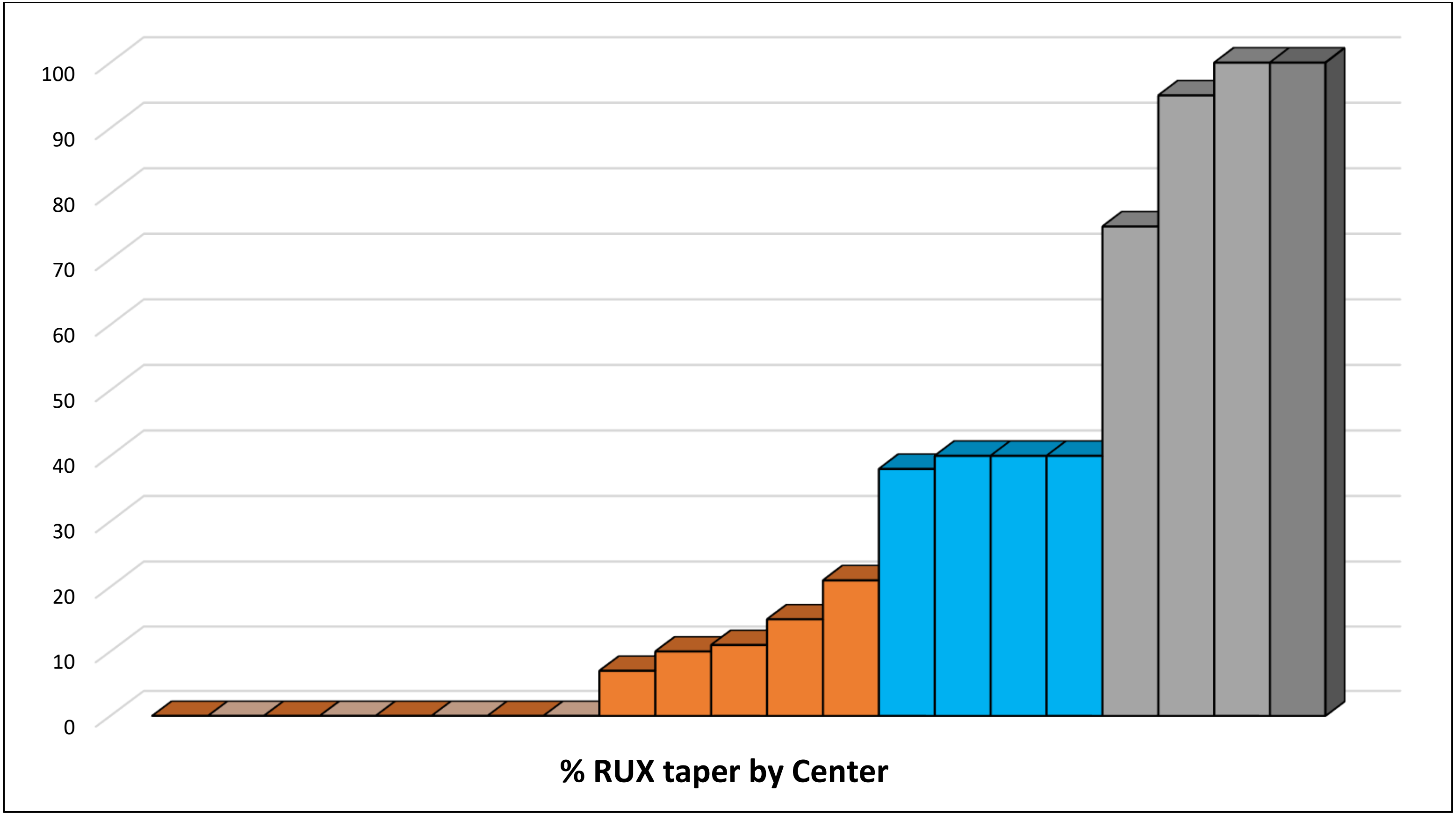

Supplement: Supplementary file 2 — Supplemental Figure 1 [file 41408_2020_392_MOESM2_ESM.pdf]
