# Supplementary material for: Ruxolitinib discontinuation syndrome: incidence, risk factors, and management in 251 patients with myelofibrosis
Source: Blood Cancer J. 2021 Jan 7;11(1):4. doi: 10.1038/s41408-020-00392-1 (PMC7791065; doi:10.1038/s41408-020-00392-1)

Supplemental Figure 2. Outcome of the three patients who experienced a severe ruxolitinib discontinuation syndrome (RDS). ICU: intensive care unit. ARDS: acute respiratory distress syndrome.

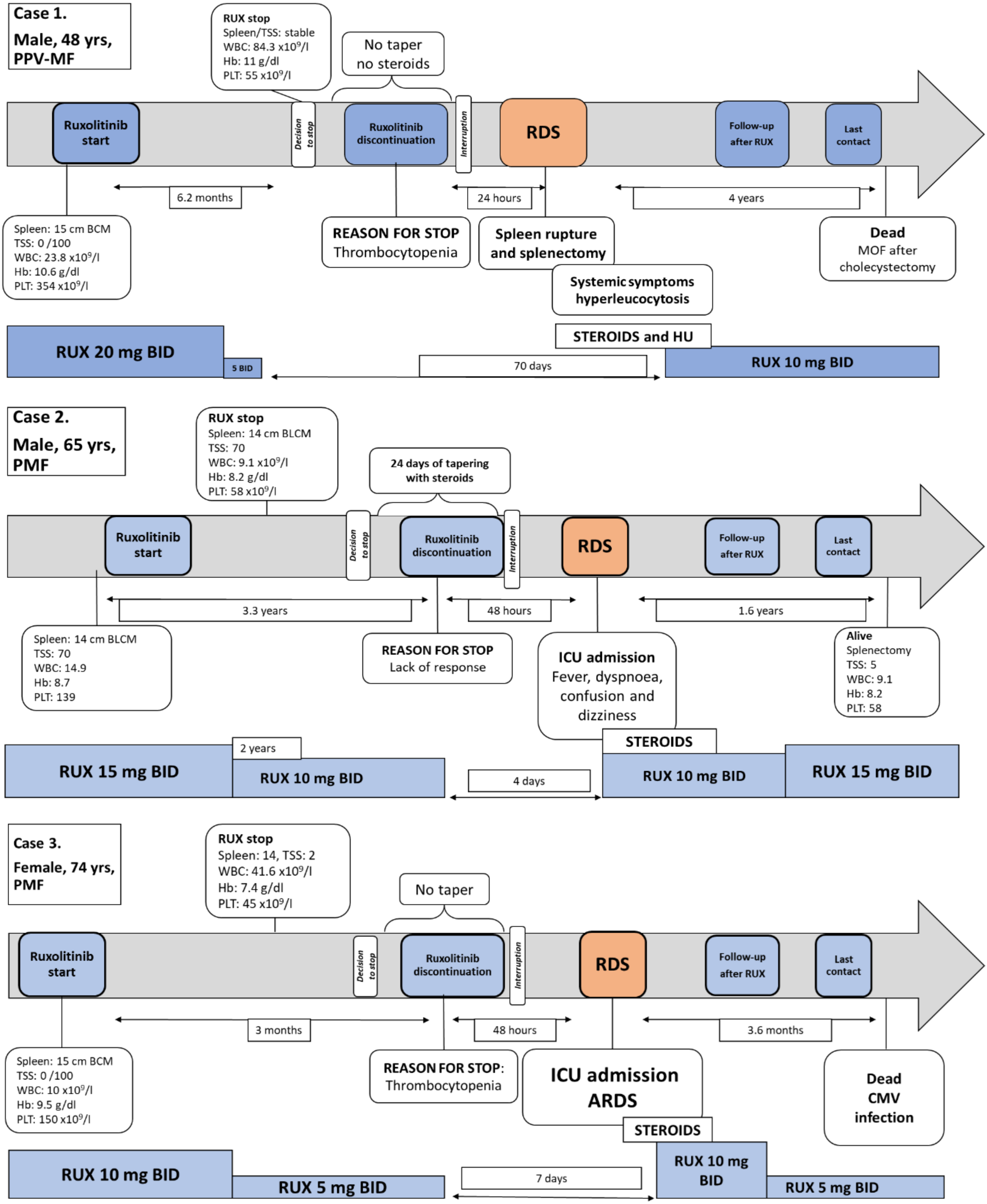

Supplement: Supplementary file 3 — Supplemental Figure 2 [file 41408_2020_392_MOESM3_ESM.pdf]
